# Distribution of virulence genes and SCC*mec* types among methicillin-resistant *Staphylococcus aureus* of clinical and environmental origin: a study from community of Assam, India

**DOI:** 10.1186/s13104-021-05473-3

**Published:** 2021-02-10

**Authors:** Deepshikha Bhowmik, Shiela Chetri, Bhaskar Jyoti Das, Debadatta Dhar Chanda, Amitabha Bhattacharjee

**Affiliations:** 1 Department of Microbiology, Assam University, Silchar, Assam India; 2 Department of Microbiology, Silchar Medical College and Hospital, Silchar, Assam India

**Keywords:** Virulence genes, SCC*mec*, Sequence types (STs), Methicillin resistant *Staphylococcus aureus*, MLST

## Abstract

**Objective:**

This study was designed to discover the dissemination of virulence genes in Methicillin-resistant *Staphylococcus aureus* from clinical, community and environmental settings**.**

**Results:**

This study includes 1165 isolates collected from hospital, community and environmental settings**.** Among them sixty three were confirmed as MRSA with varied SCC*mec* types viz; type I, type II, type III, type IV, type V, type VI, type VII, type VIII and type XII. The virulence gene such as *sea* (n = 54), *seb (n* = 21), *eta (n* = 27), *etb (n* = 2), *cna (n* = 24), *ica* (n = 2) and *tst* (n = 30) was also revealed from this study. The study underscores coexistence of resistance cassette and virulence genes among clinical and environment isolates which is first of its kind from this part of the world.

## Introduction

Incidence of community associated as well as hospital acquired *Staphylococcus aureus* has spread its infections over last few decades. *S. aureus* has the ability to cause a wide variety of infections ranging from septicaemia to toxic shock syndrome [1]. In 2011, a study from a high altitude area of Northeast India showed 18.4% MRSA among clinical specimen and carrier screening samples [2]. Another study from Northeast India found 47% of MRSA, of which 63.8% were hospital acquired and 36.2% were community acquired [3]. A previous study from our research laboratory showed 21.8% occurrence rate of MRSA from Hospital settings [4]. Rich diversification in mobile genetic element Staphylococcus cassette chromosome *mec* (SCC*mec*) leads to the expansion of antibiotic resistance determinants as well as virulence factors, which is naturally designed for the stable maintenance of the core genome environment. These virulence determinants are the genes that facilitate the successful colonisation and endurance of the organism or interrupt with the defence system of the host. [5] The dissemination of SCC*mec* types and along with virulence determinants in Methicillin Resistant *S.aureus* isolates among the hospital as well as environmental settings constitute a vast reservoir for potential spread of infection. It is important to study isolates from hospital, community and environment as this would give an insight of potential transmission route from hospital-community-environment and vice-versa. In India, SCC*mec* III, IV and V types were reported in 2010 from Mumbai within hospital and community acquired MRSA strains (ST239, ST22 and ST772). SCC*mec* types IV & V were related to community associated MRSA [6]. In a recent study from Chennai, SCC*mec* types I, III, IV, V were detected of which SCC*mec* type V was predominant [7]. The study showed 7.17% prevalence rate of MRSA in community settings whereas 81.67% from hospital environment [7]. Accumulation of toxins, virulence factors, surface proteins and enzymes have became an attributing factor of *S. aureus* along with the rapid development of multidrug resistance. Most of the *S.aureus’* pathogenicity depends on the surface proteins which help in attachment and colonisation, while the cellular protein, proteases and toxins helps in inhibiting phagocytosis thus leaving immune response ineffective [1]. The enterotoxins in *S.aureus* are most commonly found toxin associated with staphylococcal food poisoning which is regulated by two enterotoxin genes, *sea* and *seb* [8]. A serine protease enzyme such as exfoliative toxin harbouring gene *eta* and *etb* which is associated with the loss of keratinocytes, cell–cell adhesion, blister formation etc. [9]. Collagen binding adhesion (CNA) which is a virulence factor belongs to the microbial surface component recognizing adhesive matrix molecule (MSCRAMM) adhesions family, which functions in adhesion to the molecules regulated by *cna* gene [10]. Biofilm is generally associated with antimicrobial resistance. The development of biofilm is synthesised by Intercellular adhesion cluster harbouring *ica* gene [11]. Virulence factor, toxic shock syndrome toxin (TSST) is an exoprotein that belongs to pyrogenic toxin super antigen family (PTAgs) which is encoded by *tst* gene. The presence of which affects the cells’ immune system which eventually leads to death [12]. Lastly, the virulence factor fibronectin protein encoded by two genes *fnbA* and *fnbB plays a* very important role in adhesion to cells as well as also favours internalization by cells which leads to intercellular persistence and chronic staphylococcal infections [13]. So far no study had been carried out to explore the detail in involvement of virulence gene in *S.aureus* in this study area. Therefore, a comparative analysis on prevalence of virulence genes in the Methicillin Resistant *S. aureus* isolates among hospital, community as well as environmental setting is undertaken in the present study.

## Main text

### Methodology

#### Study location

Isolates were collected from hospital, community and environment from three districts of Southern Assam, India. A total of 1165 isolates were collected (550 isolates from hospital and 329 isolates from community settings and 286 isolates from the environmental setting) for the study. Clinical isolates were collected from the patient with wound infections, UTI, Post surgical infections from various wards and those visited the clinic of Silchar Medical College and Hospital, Assam, India while the environmental samples were collected from the soil (90 samples from 30 different sites), water (50 samples from 10 different sites) and sewage water (146 samples from 40 different sites) of the study area for a period of one year from June 2017 to May 2018.

#### Identification and characterization of Isolates

Isolates were cultured on Mannitol Salt broth for specifying the growth of Staphylococcal isolates and incubated for 24 h for observing the visible growth of the organism. Organisms are then spread onto MRSA Chrom agar, Baird Parker agar (Himedia) and the plates were incubated overnight. The DNA of the isolates was extracted by using the phenol chloroform method [14]. The presumptive *S. aureus* isolates were confirmed through 16 s rDNA sequence analysis. Additionally phenotypic characterization was performed through gram staining, rapid coagulase (Rapid Hi*aureus* Coagulase confirmation kit, Himedia) and latex agglutination testing (HiStaph™ Latex Test Kit, Himedia). Screening of the methicillin resistance was performed by cefoxitin agar disk diffusion and further genotypically confirmed by *coa, nuc, femB, mecA* and *mecC* PCR [15–19]. The reaction conditions of the following genes are described in Table 1.

**Table 1:** Primers: Virulence genes of *Staphylococcus aureus*

Serial no.	Primer pairs/ Target gene	Sequence	Length (bp)	Reaction conditions	Reference
1	icaA F	GACCTCGAAGTCAATAGAGGT	814bp	Initial denaturation-94°C/2min $$ \left. \begin{aligned} {\text{Denaturation}} - { 94}^\circ {\text{C}}/ 30{\text{ s}} \hfill \\ {\text{Annealing }} - { 46}^\circ {\text{C}}/ 1 {\text{ min}} \hfill \\ {\text{Extension}} - { 72}^\circ {\text{C}}/ 1 {\text{ min}} \hfill \\ \end{aligned} \right\}35\;{\text{cycles}} $$ Final Extension- 72°C/ 7 min	[31]
	icaA R	CCCAGTATAACGTTGGATACC			
2	sea F	TTGGAAACGGTTAAAACGAA	120bp		[27]
	sea R	GAACCTTCCCATCAAAAACA			
3	seb F	TCGCATCAAACTGACAAACG	478bp		[27]
	seb R	GCAGGTACTCTATAAGTGCC			
4	eta F	CTAGTGCATTTGTTATTCAA	119bp	Initial denaturation-94°C/2min $$ \left. \begin{aligned} {\text{Denaturation}} - { 94}^\circ {\text{C}}/ 30{\text{ s}} \hfill \\ {\text{Annealing }} - { 46}^\circ {\text{C}}/ 1 {\text{ min}} \hfill \\ {\text{Extension}} - { 72}^\circ {\text{C}}/ 1 {\text{ min}} \hfill \\ \end{aligned} \right\}32\;{\text{cycles}} $$ Final Extension- 72°C/ 7 min	[27]
	eta R	TGCATTGACACCATAGTACT			
5	etb F	ACGGCTATATACATTCAATT	200bp		[27]
	etb R	TCCATCGATAATATACCTAA			
6	tst F	ACCCCTGTTCCCTTATCATC	326bp		[30]
	tst R	TTTTCAGTATTTGTAACGCC			
7	cna F	GTCAAGCAGTTATTAACACCAGAC	423bp	Initial denaturation-95°C/2min $$ \left. \begin{aligned} {\text{Denaturation}} - { 95}^\circ {\text{C}}/ 30{\text{ s}} \hfill \\ {\text{Annealing }} - { 50}^\circ {\text{C}}/ 1 {\text{ min}} \hfill \\ {\text{Extension}} - { 72}^\circ {\text{C}}/ 1 {\text{ min}} \hfill \\ \end{aligned} \right\}32\;{\text{cycles}} $$ Final Extension- 72°C/ 7 min	[32]
	cna R	AATCAGTAATTGCACTTTGTCCACTG			
8	fnbA F	GTGAAGTTTTAGAAGGTGGAAAGATTG	643bp		[33]
	fnbA R	GCTCTTGTAAGACCATTTTTCTTCAC			
9	femB F	TTACAGAGTTAACTGTTACC	651bp	Initial denaturation-95°C/2min $$ \left. \begin{aligned} {\text{Denaturation}} - { 95}^\circ {\text{C}}/ 30{\text{ s}} \hfill \\ {\text{Annealing }} - { 44}^\circ {\text{C}}/ 1 {\text{ min}} \hfill \\ {\text{Extension}} - { 72}^\circ {\text{C}}/ 1 {\text{ min}} \hfill \\ \end{aligned} \right\}35\;{\text{cycles}} $$ Final Extension- 72°C/ 7 min	[29]
	femB R	ATACAAATCCAGCACGCTCT			
10	nuc F	GCGATTGATGGTGATACGGTT	270bp	Initial denaturation-95°C/2min $$ \left. \begin{aligned} {\text{Denaturation}} - { 95}^\circ {\text{C}}/ 30{\text{ s}} \hfill \\ {\text{Annealing }} - { 49}^\circ {\text{C}}/ 1 {\text{ min}} \hfill \\ {\text{Extension}} - { 72}^\circ {\text{C}}/ 1 {\text{ min}} \hfill \\ \end{aligned} \right\}35\;{\text{cycles}} $$ Final Extension- 72°C/ 7 min	[28]
	nuc R	AGCCAAGCCTTGAACGAACTAAAGC			

#### Antibiotic susceptibility testing and minimum inhibitory concentration

Kirby-Bauer disk diffusion method was used for susceptibility testing against different groups of antibiotic viz. ciprofloxacin (5 µg/ml), gentamicin (10 µg/ml), co-trimoxazole (25 µg/ml), erythromycin (10 µg/ml), clindamycin (10 µg/ml), doxycycline (10 µg/ml), and tetracycline (30 µg/ml) (Hi-media, Mumbai) and the results were interpreted according to the CLSI recommendation. *Staphylococcus aureus* ATCC 25,923 was used as control. While minimum inhibitory concentration testing was performed for oxacillin (Himedia), vancomycin, linezolid and teicoplanin as per CLSI recommendations [20].

#### Determination of virulence factor by multiplex PCR assay

Genes encoding virulence factors such as *sea* (enterotoxinA), *seb* (enterotoxinB), *eta* (exfoliative toxin A), *etb* (exfoliative toxin B), *cna* (collagen binding adhesion), *fnb* (fibronectin binding protein), *ica* (intercellular adhesion protein), *tst* (toxic shock syndrome) were studied by multiplex PCR amplification for all the MRSA isolates. Virulence specific primers were used for the study (Table 1). Three sets of multiplex PCR were employed for the study which was initiated with the 25 μl of the reaction mixture which includes (12.5 μl of the Go green Taq mixture (Promega, India), 10 pmol of each primer, nuclease free water and 50 pmol of the DNA product) with varied reaction conditions, the details of which is given in Table 1.

#### Distribution of toxin encoded virulence genes among SCC*mec* types:

SCC*mec* typing was done for the isolates by a set of three multiplex PCR assays. The details of the assays performed are as described in the previous study [21].

#### Multilocus sequence typing of SCC*mec* types carrying Virulence genes:

To establish the relatedness of SCC*mec* types with the virulence factors, amplification of MLST gene was performed according to Enright et al*.* 2000 for all the MRSA isolates carrying SCC*mec* types in combination with virulence gene. Seven housekeeping genes were targetted for the MLST study and the primers were used for amplification by targeting the genes viz; *arcC, aroE, glp, gmk, pta, tpi* and *yqiL*. The sequence were obtained and analysed in Centre for Genomic Epidemiology MLST 2.0 website (https://cge.dtu.dk/services/MLST-2.0) [22].

### Result

**Fig. 1:**
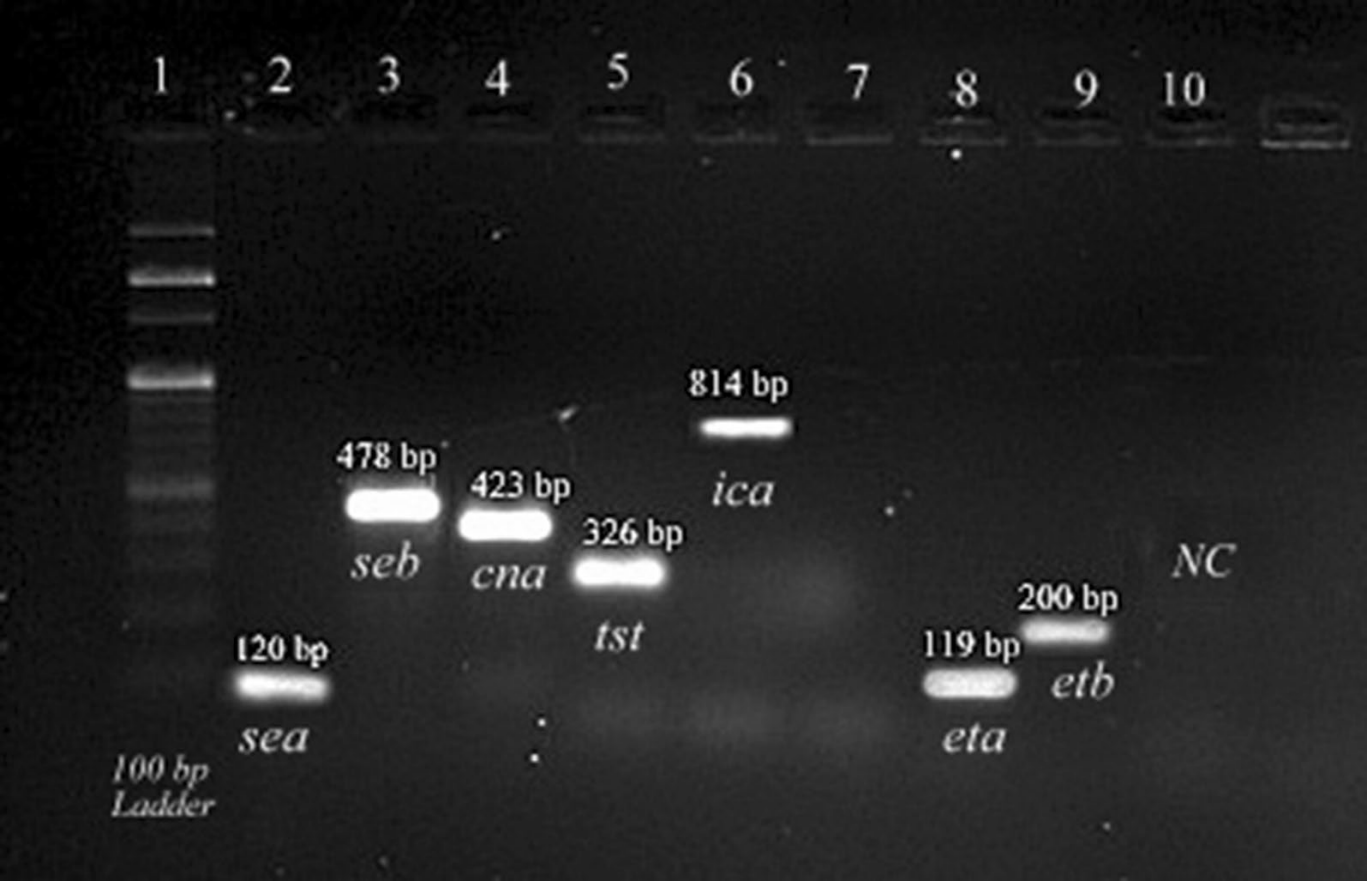
Validation and application of Methicillin resistance *Staphylococcus aureus* harbouring virulence gene Lane 1, 100 bp ladder; Lane 2, enterotoxin A *(sea* gene);lane 3, enterotoxin B (*seb* gene); lane 4, collagen binding adhesion (*cna* gene); lane 5, Toxic shock syndrome (*tst* gene); lane 6, intercellular adhesion (*ica* gene); lane 8, exfoliative A (eta gene); while lane 9, exfoliative B (etb gene), and lane 10 negative control

Among 1165 samples collected from different location, 330 staphylococcal isolates were isolated, of which 123 isolates were confirmed as *S. aureus.* Further, presence of *fem*B and *mec*A genes was also observed and sixty three isolates exhibited methicillin resistance. Antibiotic susceptibility testing showed linezolid (66.6%) was the most effective one followed by minocycline (55.5%) and doxycycline (40%). SCC*mec* typing showed that majority of the isolates associated with virulence genes were of SCC*mec* type V (33.33%), followed by SCC*mec* type II (23.08%) and SCC*mec* type VII (20%), while less number of isolates were associated with SCC*mec* type III (7.93%), type IVa (1.58%), typeVI (3.17%), type VIII (3.17%) and type XII (4.76%). (Additional file 1: Table: S2). Nine different STs of isolates with SCC*mec* types were observed where isolates of SCC*mec* type I, type II, and type VI belonged to ST 2472, ST 2039 and ST 2459 and isolates carrying SCC*mec* type IV, SCC*mec* type III were found associated with ST1551 and ST2302 while ST672, ST 5152 and ST2884 were observed with SCC*mec* type V, SCC*mec* type VII, type VIII and type XII respectively. Presence of different virulence genes were noted where 43 isolates carried enterotoxin A (*sea*) gene, 7 isolates were harbouring enterotoxin B (*seb*) gene while 15 isolates found carrying both the enterotoxin A and B gene. Exfoliative toxin gene *eta* was found in 26 isolates and *etb* gene in one while one isolate was harbouring both the exfoliative genes *eta* and *etb*. Collagen binding adhesion (*cna)* gene was found in 24 isolates, and presence of toxic shock syndrome toxin (*tsst)* gene was observed in 30 isolates. Intercellular adhesion protein (*ica*) were found to be present in 2 isolates which is shown in Fig. 1. It was observed that enterotoxin A (*sea*) gene (92.06%) was the most predominant staphylococcal toxin followed by toxic shock syndrome toxin (*tst*) (50.79%), exfoliative toxin A (*eta*) gene (47.61%), *cna* (38.09%), *seb* (33.33%) and *ica* and *etb* gene (3.1%). It was also observed that, environmental sources acted as reservoir as majority of the *sea* gene (47.6%) was prevailing, followed by *tst* (28.57%) and *cna* (26.98%) (Table 2).

**Table 2:** Prevalence of *S. aureus* virulence genes among different settings

Virulence Genes	No. of Isolates (n = 63)		
	Hospital	Community	Environment
*sea*	15 (23.8%)	13 (20.6%)	30 (47.6%)
*seb*	4 (0.63%)	5 (0.79%)	12 (19.04%)
*eta*	4 (0.63%)	11 (17.46%)	13 (20.63%)
*etb*	1 (0.16%)	-	1 (0.16%)
*ica*	1 (0.16%)	1 (0.16%)	-
*cna*	4 (0.63%)	3 (0.47%)	17 (26.98%)
*tst*	5 (0.79%)	9 (1.42%)	18 (28.57%)

sea, Enterotoxin A; seb, Enterotoxin B; eta, Exfoliative toxin A; etb, Exfoliative toxin B; ica, Intercellular adhesion protein; cna, Collagen binding protein; tst, toxic shock syndrome toxin

The MRSA isolates having combination of the virulence genes were also focused in this study and it was found that twenty nine different combination of virulence genes coexisted. Moreover it was also observed from the current study that three isolates were having a combination of six virulence genes, seven isolates with a combination of five virulence genes, and thirteen isolates showed different combinations with four virulence genes whereas combination three virulence genes were observed in 19 isolates. (Additional file 1: Table S1).

### Discussion

Virulence factors play a key step for pathogenic invasion leading to staphylococcal infection in the hospital, community and in environmental settings. Rich diversity of virulence associated genes within *S. aureus* is reported worldwide [23, 24]. It was observed in the present study that the virulence determinants such as *sea, tst* and *eta* gene were found to be more predominant in the study isolates. However, in an earlier study conducted by Mojtabi et al. 2018, reported high prevalence of *eta, etb* and *etd* genes in *S.aureus* among clinical isolates [25]. In another study by Wu et al. 2011 and van Trijp et al. 2010, low prevalence of *eta* gene was found as compared to our study [26, 27]. Studies from different countries revealed that *sea* is the most common enterotoxin recovered followed by *seb* and *sed* along with different Staphylococcal enterotoxins (SE’s) associated with outbreaks [28]. Studies from Korea by Lim et al. 2010, stated that Staphylococcal enterotoxin (*seg, sei, sec*) genes and toxic shock syndrome toxin (*tst*) genes were most common in clinical MRSA isolates [29]. Thus from the above study it can be explained that the high frequency of exfoliative toxin (*eta*) which facilitated colonization and invasion may be due to wound infection and presence of enterotoxin pose a high risk to food borne intoxication. A recent study by Kozajdi et al. 2019, were carried out in hospital and environmental settings such as large scale animal breeding, waste water treatment plants etc. [30]. Also Study by Boopathy et al. 2017 and Friese et al. 2013 carried out their work on the people exposed environmentally such as waste water treatment plants, animal farms etc. [31, 32] A study by Kumar et al. 2009 have detected four virulence genes viz; cna (16 isolates), icaA (19 isolates), hlg (21 isolates), and sdrE (18 isolates) from the paper currency collected from mutton shops, vegetables shop, snacks stall, restaurants etc. [1] whereas in our current study six virulence genes viz. *sea* (30 isolates), *tst* (18 isolates), *cna* (17 isolates) *eta* (13 isolates) and *seb* (12 isolates) were detected from the environmental settings such as soil, water and sewage.

Our study involved MRSA targeting virulence genes and showed 31% of the occurrence rate which was comparatively lower when compared to the results observed by Liang et al. 2019 (55%) and Koosha et al. 2013 (87.6%) [33, 34]. Current study observed sixty three MRSA isolates of which 85.71% of the isolates were found to harbour enterotoxin *A* (*sea*) gene. This rate is found to be higher than the previous studies [35, 36]. A study from Mumbai, India in 2010 reported the presence of SCC*mec* types III, IV & V within hospital & community acquired MRSA strains (ST 239, ST22 & ST772) while SCC*mec* types IV & V were related to Community acquired MRSA [6]. Also in a recent study from Chennai, reported the presence of SCC*mec* types I, III, IV & V of which SCC*mec* type V was predominant [7]. This study also showed high prevalence rate of MRSA (81.67%) in the hospital environment as compared to community (7.17%). While our study from Northeastern part of India detected the presence of SCC*mec* types I, II, III, IVa, V, VI, VII, VIII & XII from hospital, community and environmental settings. Another study by Carvalho in 2019, from Brazil has detected SCC*mec* types I, IVa, & V and virulence genes from Intensive Care Unit (ICU), equipment surfaces and healthy children [37]. Mobile genetic element (SCC*mec*) carries both resistance as well as virulence genes. It has been found from the study of Lim et al. 2010 that 97.6% of SCC*mec* type II carried *sec, seg, sei* and *tst* gene; 73% of SCC*mec* type III strains carried *sea* gene and 89.7% of SCC*mec* type IV strain carried *sec, seg, sei* genes [29]. While our study reveals that 28.2% of SCC*mec* type II isolates carried *sea, seb, cna, tst and eta* genes, 10% of SCC*mec* type III carried *sea, seb, cna* and *tst* gene, 34.1% of SCC*mec* type V was found to carry *sea, seb, ica, cna, tst eta* and *etb* genes and 20% of SCC*mec* type VII carried *sea, seb, cna, tst, eta* and *etb* genes. However the rest isolates with SCC*mec* types I, IV, VI, VIII and XII showed less prevalence rate as compared to the above. The study conducted by Liang et al. 2016 showed that ST239-SCC*mec*AIII-t37 clone was more prevalent one as reported from china whereas our current study showed that ST2884-SCC*mec* type V was more predominant than the other sequence type [33].

This study involved a comparative analysis of virulence genes found to be prevailing in hospital, community as well as in environmental settings. It was recorded that majority of the isolates containing virulence gene were found in the environmental sources which is in contrast to the other studies where virulence genes were observed in clinical settings [23, 34] and underscores the risk of acting environmental sources as reservoir.

## Limitations

This study warrants a proteomic approach to analyse the mode of transfer of virulence gene which may be from environment setting to the hospital and community origin and vice versa.

## Supplementary Information

**Additional file 1: Table S1.** General Characteristics of 63 methicillin-resistance* Staphylococcus aureus* isolates from hospital –community- environment in Southern Assam, India. **Table S2.** SCCmec types and their origin.

## Data Availability

All the data generated in this research work are presented in this research article. In case of any additional information requirement corresponding author will be providing the necessary information as per ethical guidelines.

## Abbreviations

SCC*mec*: Staphylococcal cassette chromosome *mec*
MLST: Multi-locus sequence typing
MRSA: Methicillin resistant *Staphylococcus aureus*
