# Supplementary material for: Distribution of virulence genes and SCCmec types among methicillin-resistant Staphylococcus aureus of clinical and environmental origin: a study from community of Assam, India
Source: BMC Res Notes. 2021 Feb 10;14:58. doi: 10.1186/s13104-021-05473-3 (PMC7876809; doi:10.1186/s13104-021-05473-3)
Supplement: Supplementary file 1 — Additional file 1: Table S1. General Characteristics of 63 methicillin-resistance Staphylococcus aureus isolates from hospital –community- environment in Southern Assam, India. Table S2. SCCmec types and their origin. [file 13104_2021_5473_MOESM1_ESM.docx]

**Supplementary Table**

**Table S1: General Characteristics of 63 methicillin-resistance *Staphylococcus aureus* isolates from hospital –community- environment in Southern Assam, India.**

| Sl. No. | Isolates number | Origin | Isolation Source | MRSA origin | SCC*mec* type | Virulence gene profile |
| --- | --- | --- | --- | --- | --- | --- |
| 1. | PM1 | **E1** | **Sewage** | **EA** | **I** | *sea, seb, cna, tst ,eta* |
| 2. | KS1 | **E1** | **Soil** | **EA** | **II** | *sea, seb, eta, cna,tst* |
| 3 | MR1 | **E1** | **Soil** | **EA** | **II** | *sea, cna, tst* |
| 4 | MSV7 | **E1** | **Sewage** | **EA** | **II** | *sea, seb* |
| 5 | 1243 | **C1** | **Urine** | **CA** | **II** | *eta, tst, cna* |
| 6 | 0094 | **C1** | **Urine** | **CA** | **II** | *sea, eta, tst* |
| 7 | 2/13 | **C1** | **Urine** | **CA** | **II** | *Sea, eta,* |
| 8 | 2380 | **H1** | **Urine** | **HA** | **II** | *sea, tst, seb* |
| 9 | 0295 | **H1** | **Urine** | **HA** | **II** | *sea* |
| 10 | 3165 | **H1** | **Pus** | **HA** | **II** | *sea, eta* |
| 11 | 1314 | **C1** | **Urine** | **CA** | **II** | *Sea, eta, tst* |
| 12 | 3243 | **H1** | **Pus** | **HA** | **II** | *seb, eta* |
| 13 | 5716 | **C1** | **Urine** | **CA** | **II** | *sea, seb, eta, tst* |
| 14 | 5725 | **C1** | **Pus** | **CA** | **II** | *Sea, seb, tst* |
| 15 | 1491 | **C1** | **Urine** | **CA** | **II** | *sea, tst, eta,* |
| 16 | 0166 | **C1** | **Urine** | **CA** | **II** | *sea, seb, cna,tst,eta* |
| 17 | 1002 | **H1** | **Urine** | **HA** | **III** | *sea* |
| 18 | 4004 | **H1** | **Sputum** | **HA** | **III** | *sea* |
| 19 | 4199 | **H1** | **Tissue Fluid** | **HA** | **III** | *sea, tst* |
| 20 | MSMC1 | **E1** | **Water** | **EA** | **III** | *sea, seb, cna, tst* |
| 21 | MSV4 | **E1** | **Soil** | **EA** | **III** | *Sea, eta, tst, cna* |
| 22 | 4283 | **C1** | **Pus** | **CA** | **IVa** | *sea, eta, tst* |
| 23 | 106 | **H1** | **Urine** | **HA** | **V** | *sea, seb, cna* |
| 24 | 1979 | **C1** | **Urine** | **CA** | **V** | *sea, eta, tst* |
| 25 | 1/18 | **H1** | **Urine** | **HA** | **V** | *sea,* |
| 26 | 5423 | **H1** | **Urine** | **HA** | **V** | *sea, cna* |
| 27 | 3287 | **H1** | **Pus** | **HA** | **V** | *sea* |
| 28 | 4181 | **H1** | **Blood** | **HA** | **V** | *Sea, eta* |
| 29 | 3083 | **H1** | **Urine** | **HA** | **V** | *Sea, cna* |
| 30 | 4184 | **H1** | **Pus** | **HA** | **V** | *Sea, eta* |
| 31 | 4142 | **H1** | **Blood** | **HA** | **V** | *sea* |
| 32 | 4285 | **H1** | **Nasal Swab** | **HA** | **V** | *Sea, eta* |
| 33 | 3173 | **H1** | **Ear Swab** | **HA** | **V** | *sea, tst* |
| 34 | 3289 | **H1** | **Pus** | **HA** | **V** | *sea* |
| 35 | 3243 | **H1** | **Blood** | **HA** | **V** | *seb* |
| 36 | 3132 | **H1** | **Blood** | **HA** | **V** | *sea* |
| 37 | 1/13 | **C1** | **Pus** | **CA** | **V** | *seb, ica, eta* |
| 38 | 4/11 | **H1** | **Urine** | **HA** | **V** | *Sea,eta* |
| 39 | 3039 | **H1** | **Urine** | **HA** | **V** | *etb,ica* |
| 40 | 4943 | **C1** | **Pus** | **CA** | **V** | *sea,cna, eta* |
| 41 | 1863 | **H1** | **Aural swab** | **HA** | **V** | *Sea, tst* |
| 42 | 3/20 | **C1** | **Urine** | **CA** | **V** | *sea, tst, eta* |
| 43 | 3/29 | **C1** | **Urine** | **CA** | **V** | *Sea,eta* |
| 44 | MSV1 | **E1** | **soil** | **EA** | **VI** | *Sea, seb, cna, tst* |
| 45 | MSV8 | **E1** | **soil** | **EA** | **VI** | *Sea, seb, cna* |
| 45 | NM1 | **E1** | **Water** | **EA** | **VII** | *Sea, eta, tst* |
| 46 | JM2 | **E1** | **Water** | **EA** | **VII** | *etb* |
| 47 | 1425 | **C1** | **Urine** | **CA** | **VII** | *Sea, eta* |
| 48 | 2890 | **H1** | **Nasal Swab** | **HA** | **VII** | *sea* |
| 49 | MH6 | **E1** | **Sewage** | **EA** | **VII** | *sea, cna, seb, tst* |
| 50 | MR6 | **E1** | **sewage** | **EA** | **VII** | *sea, cna,eta* |
| 51 | MSV8 | **E1** | **Soil** | **EA** | **VII** | *Sea, tst,cna* |
| 52 | MG14 | **E1** | **water** | **EA** | **VII** | *Sea, tst, seb* |
| 53 | MD14 | **E1** | **sewage** | **EA** | **VII** | *Sea, cna,seb, tst* |
| 54 | MSMC33 | **E1** | **Water** | **EA** | **VII** | *Sea, cna, tst* |
| 55 | M G9 | **E1** | **water** | **EA** | **VII** | *Sea, seb, cna, tst* |
| 56 | SS39 | **E1** | **Soil** | **EA** | **VII** | *Sea, cna, tst* |
| 57 | RM1 | **E1** | **soil** | **EA** | **VII** | *Sea, eta , seb* |
| 58 | CMH1 | **E1** | **sewage** | **EA** | **VIII** | *Sea, cna, eta, tst* |
| 59 | MSMC20 | **E1** | **sewage** | **EA** | **VIII** | *Sea, cna, seb, tst* |
| 60 | MSMC17 | **E1** | **Sewage** | **EA** | **XII** | *Sea, tst,seb, cna* |
| 61 | 1350 | **H1** | **Urine** | **HA** | **XII** | *Sea, tst, eta ,cna* |
| 62 | 7696 | **H1** | **Urine** | **HA** | **XII** | *Sea, seb* |
| 63 | 5090 | **H1** | **Throat swab** | **HA** | **Not identified** | *Sea, seb* |

**Note: E1, Environmental origin; C1, community origin; H1, Hospital origin; EA, Environmental –associated MRSA; CA, Community-associated MRSA; HA, Hospital –associated MRSA**

**Table S2: SCC*mec* types and their origin**

| SCC*mec* types | Number of Isolates | Percentage | Origin |
| --- | --- | --- | --- |
| I | **1** | **1.58%** | **Environment -1** |
| II | **15** | **23.80%** | **Hospital-7**  **Community-5**  **Environment-3** |
| III | **5** | **7.93%** | **Hospital-2**  **Community-1**  **Environment-2** |
| IV | **1** | **1.58%** | **Hospital-1** |
| V | **21** | **33.33%** | **Hospital-12**  **Community-9** |
| VI | **2** | **3.17%** | **Environment-2** |
| VII | **13** | **20%** | **Hospital-2**  **Community-1**  **Environment-10** |
| VIII | **2** | **3.17%** | **Environment-2** |
| XII | **3** | **4.76%** | **Hospital-2**  **Environment-1** |
| Total | **63** |  |  |
